# Child Farm-Related Injury in Australia: A Review of the Literature

**DOI:** 10.3390/ijerph18116063

**Published:** 2021-06-04

**Authors:** Jessie Adams, Alison Kennedy, Jacqueline Cotton, Susan Brumby

**Affiliations:** 1 School of Medicine, Deakin University, 75 Pigdons Road, Waurn Ponds, VIC 3216, Australia; alison.kennedy@wdhs.net (A.K.); jacquie.cotton@wdhs.net (J.C.); susan.brumby@wdhs.net (S.B.); 2 National Centre for Farmer Health, Western District Health Service, Hamilton, VIC 2200, Australia

**Keywords:** child, farms, child health, workplace, agriculture, risk factors, humans, occupational health, farm children, child development

## Abstract

Children on farms have been identified as a population vulnerable to injury. This review seeks to identify child farm-related injury rates in Australia and to determine the key hazards and contributing risk factors. This critical review utilised the PRISMA guidelines for database searching. Research from the year 2000 onward was included as well as earlier seminal texts. Reference lists were searched, and the relevant research material was explored. Our primary focus was on Australian peer-reviewed literature with international and grey literature examples included. Evidence suggests that there is limited Australian research focusing on child farm-related injuries. Child representation in farm-related injuries in Australia has remained consistent over time, and the key hazards causing these injuries have remained the same for over 20 years. The factors contributing to child rates of farm injury described in the literature include child development and exposure to dangerous environments, the risk-taking culture, multi-generational farming families, lack of supervision, child labour and lack of regulation, limited targeted farm safety programs, underuse of safe play areas, financial priorities and poor understanding and operationalisation of the hierarchy of control. It is well known that children experience injury on farms, and the key hazards that cause this have been clearly identified. However, the level of exposure to hazards and the typical attitudes, behaviours and actions of children and their parents around the farm that contribute to chid injury remain unexplored.

## 1. Introduction

Globally, injuries are a leading cause of death and disability among children [1]. Unintentional injuries have replaced infectious diseases as the most serious child health issue in the industrialised world [2,3,4] —accounting for approximately 40% of deaths in developed countries [1] and contributing to disability, future health care needs, intellectual development delays and reduced economic and productivity prospects [5]. 

In Australia, injury is the leading cause of death and hospitalisation of children aged one to fifteen years [6,7]. Both fatality and hospitalisation rates rise with increasing remoteness. Between 2013 and 2017, the rate of fatal injuries among Australian children in outer regional, remote and very remote areas was three times higher than in major cities [6]. 

Farming families are defined as families where at least one adult is a farmer or farm manager [8]. According to the Australian Bureau of Statistics, in 2016, there were 87,325 farming families in Australia, including 48% consisting of parents with children living with them [8]. Children and adolescents on farms have been identified as vulnerable; suffering premature death, morbidity and disability from injury [1,9]. The blurred distinction between the farm as a home and a workplace means children are exposed to hazards typically not present in most homes [10,11,12,13,14,15,16,17,18]. Additionally, most farming parents want to involve their children in the unique farming lifestyle, often giving them on-farm work responsibilities at a young age [17,19]. 

This review aims to: (1) identify child mortality and morbidity rates from farm-related injuries in Australia; (2) explore the key hazards causing these injuries; and (3) understand factors that may contribute to the risk of child farm-related injury. 

## 2. Methods

**Figure 1:**
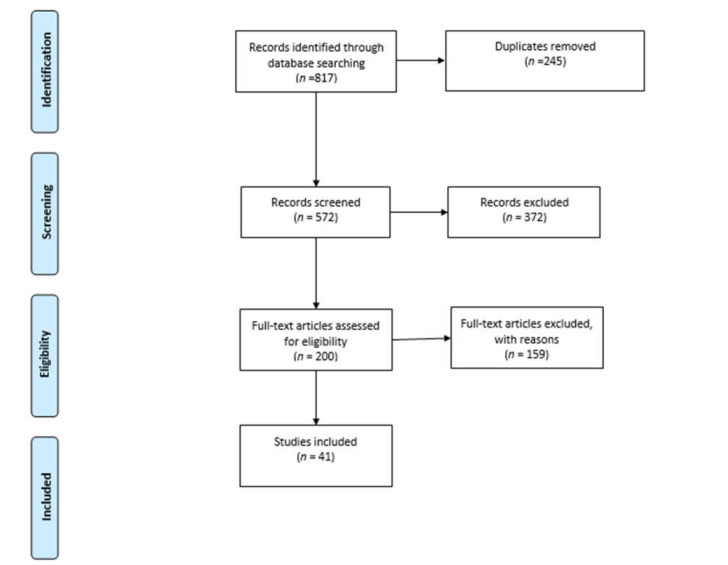
PRISMA diagram of database research publication selection [21].

This critical review identifies significant work in the field, and analyses and evaluates the quality of the existing literature on child farm injury and safety [20]. A broad and deep coverage of the topic was undertaken. We focused on an area of research that has not previously been comprehensively explored in Australia. The PRISMA guidelines were followed for the database search process [21] (Figure 1). Articles were removed during the screening and eligibility phases due to inability to find full texts, date of text, wrong population focus, wrong outcomes and wrong settings. 

Studies were included if they encompassed children aged under 18 years. A snowballing and saturation approach was then adopted, with reference lists searched for further relevant material. The key focus was on Australian peer-reviewed literature. However, given the limited recent research, grey literature and international research—particularly North American—were included to provide examples relevant to the Australian context. 

Initial search terms used were: child*, adolescen*, teenag*, paed*, farm*, agricultur*, injur*, morbidit*, mortalit*, safe*, risk* and behav*. The Boolean operators ‘AND’ and ‘OR’ were utilised to increase the search scope. The databases searched were Medline, Embase, Eric, Informit, Global Health and SafetyLit. As limited recent research has been conducted on child farm safety in Australia, literature from 2000 to 2020 and earlier seminal texts were included. Article titles and abstracts were assessed to ascertain relevance and duplicates removed [22]. Full-texts were then read to determine compatibility with other inclusion criteria:

- Research reported on human subjects
- English language
- Peer-reviewed or grey literature
- Focus on the farming context
- Focus (primary or secondary) on children on farms

## 3. Results

### 3.1. Child Farm-Related Injury Mortality and Morbidity Statistics 

#### 3.1.1. Mortality from Injury 

Mandryk and Harrison [23] first explored work-related deaths of Australian children for the period 1982–1984. They acknowledged existing workplace controls as effective in child injury prevention, with farms as the exception. Since this time, the majority of research has largely focused on specific regions or farming hazards [11,24,25,26,27,28,29]. Peachey and colleagues [30] provided the most recent literature on child farm-related fatalities across Australia. Between 2001 and 2019, 222 children under 15 years of age died from farm-related injuries. This represented 15% of all farm-related deaths, which is consistent with earlier research by Lower and colleagues [31]. It is widely acknowledged that boys make up the majority of farming fatalities, accounting for 73% of child farm deaths between 2001 and 2019 [30]. 

Peachey and colleagues [30] emphasised the persistent rate of child farm fatalities over time, with only a 0.009 reduction in deaths per year over the 18-year period. This minor reduction aligns with the trend identified by Lower and colleagues [31]; a reduction in overall farm-related fatalities in the early 1990s and then a stabilisation of rates from 2005. It has been hypothesised that the small reduction in deaths is due to the decline in farmers and consequently a decrease in children on farms [30]. This could also be due to improved treatments and emergency transport services in rural areas (i.e. injuries occur but do not result in as many fatalities). With no comprehensive research investigating non-fatal child farm-related injuries, it is challenging to determine and interpret trends over time. 

While previous literature has largely adopted a focus on the broader farming population, consistent child representation in farm fatality statistics has been reported [14,32,33,34]. Lower and Herde [14] identified 326 farm-related deaths on the National Coroners Information System between 2003 and 2006; 17% were children. Safework Australia [35] attributed 51 workplace deaths to children between 2003 and 2016. The suggestion from this data—that children only make up 7% of the 744 agriculture workplace deaths—is counter to the more targeted research previously conducted by Lower and colleagues [14] over the same period. This highlights the limitations in farm workplace death reporting and indicates that not all non-work-related fatalities that occur on farm are captured. 

Lower and Herde [14] reported that 48% of on-farm deaths between 2003 and 2006 were non-work related (bystanders or leisure activities). This even spread of work-related and non-work-related deaths highlights the blurred division between the farm as a workplace and home—with nearly all of the 55 child farm deaths between 2003 and 2006 identified as non-work-related. 

While recent work conducted by Peachey and Lower [30] has helped to fill the gap in knowledge on child farm fatalities in Australia, a clear understanding of the incidence of farm-related injuries among children is still lacking. Most existing literature focuses on overall farm or workplace deaths, often identifying children as a secondary population [24,36,37]. This lack of Australia-wide literature provides challenges in quantifying, comparing and drawing conclusions. 

#### 3.1.2. Morbidity from Injury 

Since the 1980s, children on farms have been recognised in academic literature as a population that is particularly vulnerable to farm-related injury [38]. Despite research mentioning child farm-related non-fatal injuries [39,40,41], there is yet to be comprehensive research examining this in Australia. To date, most non-fatal injury research has concentrated on the state of New South Wales, limiting its validity (and the validity of proposed prevention measures) across the wider Australian context. An Australian Institute of Health and Welfare report provided the most recent Australia-wide farm-related injury hospitalisation data [39]. Approximately 22,000 people were hospitalised from 2010–2011 to 2014–2015 due to farm-related injuries, including over 2000 (9%) children under 15 years. Although focused solely on New South Wales, between 2010 and 2014, Lower and Mitchell [41] attributed 6270 hospital admissions to farm-related injuries, of which 16.3% were children. As hospital record procedures vary, and cause and location of injury is not always reported, and an accurate picture of non-fatal farm-related injuries may not be captured. 

### 3.2. Key Hazards Causing Child Farm-Related Injury 

Farms present a diverse range of hazards to children through exposure to machinery, grain silos, farm buildings, water sources, chemicals, noise, dust, animals, heights, sun and firearms [42,43]. However, the key hazards—accounting for 75% of child fatalities—have changed little over time. These seven hazards include water bodies, quad bikes, tractors, utility vehicles (trucks), cars, motorbikes and horses [30]. 

#### 3.2.1. Water Bodies 

In Australia, drowning is the leading cause of death in children under 15 years [44]; accounting for 31% of child farm-related deaths between 2001 and 2019 [30]. This was most commonly represented by the drowning of children under four years of age in farm dams [30,45]. 

#### 3.2.2. Quad Bikes/Motorbikes 

Quad bikes (sometimes referred to as All-Terrain Vehicles (ATVs)) have emerged as a leading cause of farm-related morbidity and mortality [10,14,30,39,40,46]. Approximately 80% of Australian and New Zealand farms have at least one quad bike that is utilised daily [47]. The use of quadbikes by children on farms (residents and visitors), both as drivers and passengers, is a contentious issue due to the traumatic injuries sustained, largely due to their heavy weight and large size compared to children [48]. 

Peachey and colleagues [30] attributed over 20% of all child (under 15) fatalities on farms to quadbikes and motorbikes between 2001 and 2019. Henley and Harrison [39] described 42% of child farm-related injury hospitalisations between 2010–2011 and 2014–2015 were due to quadbike and motorcycle incidents. Specifically, young males, aged five to fourteen, are overrepresented in motorbike/quadbike injury statistics [39,46,49]. 

On farms, motorbikes and quadbikes are used for both work and recreation—this is reflected by the even split of work and recreation fatalities in Australia [50]. Children are most often represented in recreational motorbike/quadbike injuries [30,50]. Although not focused solely on farms, research conducted by both McIntosh and colleagues [50] and Amey and Christey [51] unsurprisingly identified at least half of the children injured on quadbikes were riding adult-sized bikes. It is widely acknowledged and included in the manufacturer’s guidelines that children under 16 years should not drive or be passengers on quadbikes [51]. 

#### 3.2.3. Utility Vehicles/Tractors/Cars 

Children on farms are exposed to heavy vehicles and machinery that they would otherwise not come in contact with outside the farm environment [11,14,29,30,46]. Standard vehicle safety procedures are often not adhered to when on the farm. For example, seatbelts—whilst compulsory when riding in a vehicle on Australian roads—have been identified as the least utilised child safety practice on farms [29]. Vehicle incidents on farms typically involve children riding in, or on the utility tray, riding unrestrained in the cabin, and as casualties of vehicle rollovers or low speed run-overs [30]. Although health promotion campaigns have discouraged children riding on tractors, they remain a leading cause of injury [18,42,52]. With farm machinery being expensive and the ever-present economic difficulties in the rural sector, farmers may not have the latest or safest equipment [53]. 

#### 3.2.4. Horses 

While boys are more likely to be injured by motorbikes, girls are more likely to experience injury from horses [39]. Approximately 80% of horse-related injuries occur to girls, with the most vulnerable aged 10 to 14 years [29,39,46]. 

### 3.3. Factors Contributing to Child Farm-Related Injuries 

The prevention of child farm injuries is a complex issue for both parents and safety professionals [54]. Children are vulnerable due to their physical, cognitive and behavioural characteristics [55]. Haddon’s Injury Model explains that injuries occur due to an uncontrolled interaction between a host, agent and environment. These interactions are unique in regard to child injuries as a parent/adult is usually responsible for controlling or supervising the interactions [56]. The consistent rate of farm injuries indicates that children are regularly engaging with farming hazards [54]. Typically, injury type varies according to age and developmental stage. Other potential influencing factors include farm type, season and periods of increased farm activity [57,58]. Understanding the context of injury is important for developing prevention strategies. 

Visitors to the farm are also considered vulnerable to farm-related injuries, although little research has explored this [36,43]. There is a challenge in understanding the rate of child farm visitor injuries as the number of visitors to farms is unknown [30,39]. Typically, farm visitors have less knowledge of farm hazards and risks. Peachey and colleagues [30] identified every third case of child farm-related fatality as a farm visitor; representing 21% of child farm drownings, along with 43% of quad bike, 50% of motorbike and the majority of utility fatalities [30]. 

#### 3.3.1. Risk-Taking Culture 

Australian farmers characteristically view safety as common sense, considering incidents as ‘unlucky’ rather than avoidable [16,59]. Risk-taking traits and attitudes to risk are commonly passed on through generations and attributable to geographic isolation, frequent exposure to occupational hazards, personal behaviours and the influence of cultural norms [16,60,61]. Greater risk-taking, combined with increased exposure to hazards, can result in a heightened likelihood of injury. 

A recent report by Farmsafe Australia [62] highlighted four aspects of farming culture that create barriers to improvement in farm safety; money and time pressures, belief that incidents ‘will not happen to me’, reliance on common sense and the generational transfer of farming methods and attitudes. There has been a view that little can be done to increase the health and safety of farmers, families and workers [63]. Fatalistic attitudes reflecting the inevitability of injury are thought to provide parents with perspective on the impossible task of preventing all possible harm—releasing them from overprotection and responsibility [54]. Nilsson [17] reported that parents worry everyday about what could happen to their children on the farm and that parents acknowledged they cannot protect their children at all times on the farm because they have their own farm work to complete. Conversely, Dixey [64] explored the importance of considering health promotion solutions in different contexts. What appears ‘rational’ to one culture may not to another, and people’s lack of knowledge on causes of injury can incite the belief of injury as an ‘act of God’ and fatalism. 

#### 3.3.2. Multi-Generational Farming Families 

Families play a pivotal role in child development, particularly in modelling and enforcing safety practices. Children imitate behaviours and actions around them, assuming what they see is right [1,17]. Farming families often give children responsibilities and tasks at a young age, and parents may overestimate their child’s physical, social and cognitive capacity to complete tasks [17,19,30]. Traditionally, farming methods are passed down from generation to generation with on-the-job trial and error. Limited formal farming and safety training can mean unsafe operations are perpetuated [16,63]. 

The blurred distinction between work, home, personal identity and occupation in farming can result in behaviours that are typically not acceptable within other workplaces [16]. Elliot and colleagues [54] determined that farming parents are aware of the occupational hazards for their children. However, farmers under 55 years—those most likely to be parents of dependent children—have the lowest safety scores [65]. As such, they are the least likely to demonstrate a positive example to their children. However, excluding children from participating in farming practices can be perceived as threatening to the continuation of traditions and values [54,66]. Rather, parents believe that exposure, education and knowledge about risks should equip children to be safe [54,66]. 

Internationally, migrant/seasonal workers have been identified as a population vulnerable to injury [67]. In the United States, these workers’ children often work in the fields beside them from around 12 years of age [68]. Migrant/seasonal farm workers are a key workforce in Australian agriculture, particularly in horticulture [69]. However, they are not identified in injury data, and it is unknown if their children are exposed to the farm workplace or even provide assistance to their parents. 

#### 3.3.3. Child Development and Dangerous Environments 

Children live in an environment built for adults; with little power or control and a reduced capacity to predict or respond to danger [1,42]. There is a stereotypical view that farms are healthy places for children to grow and develop and can provide an enriching life. However, the farm is also a work environment with hazards not typically present at most homes [66]. Children’s size, limited physical strength, inexperience, increasing independence, impulsivity, curiosity and immature coordination increase their injury risk. Additionally, they have poorly developed danger identification and are influenced by peer pressure [70]. Children are unable to extricate themselves from potentially deadly situations where this may be possible for adults [71]. Potential hazards and injury risks differ with a child’s stage of development:

- Toddlers—falls when playing and exploring the worksite and through interaction with animals [7].
- Young children (five to nine years)—distraction and impulsivity [18].
- Older children/adolescents (10–14 years)—performing farm tasks or during recreation; able to identify hazards and follow basic rules/procedures but are easily distracted, challenge authority, are overly confident and influenced by peers [42,72].

Child safety in the farming environment is complex—parents must consider the home, workplace, geographical isolation and their child’s cognitive development and physical ability. They also need to match child safety and ability with the needs and demands of farming life. A delicate and difficult balance is required between encouraging children to grow and develop through stimulation and inclusion in the family farm life and providing a safe environment [66,73]. According to Elliot and colleagues [54], farming parents weigh up the perceived benefits and hazards of involving their children in the farm workplace, and that when these are balanced, the benefits outweigh the risks. 

However, other research indicated that parents overstate the cognitive ability of children [17,19,30]. Recent work by Summers and colleagues [74] reported that children break rules and take risks, but also recognise that they are modelling the unsafe behaviour of adult relatives. Farming is one of the most dangerous industries for adults, and when children engage in agriculture activities, the risks of injury are increased [75]. Evidence shows the importance of parents selecting developmentally appropriate tasks for their children when engaged in farm work [2,76]. However, this can be difficult to achieve as Neufield and colleagues [77] noted that parents have a clear belief system that justifies their child farm work. They suggested any effort to reduce risks to children would need to consider the benefits parents perceive farm work gives their children and how the need for labour determines the task over their child’s maturity and development levels [77]. Nilsson [17] suggested these behaviours could be a reflection of an industry culture that values productivity over human conditions. 

#### 3.3.4. Child Labour and Lack of Regulation 

Agriculture is a priority industry in the Australian Work Health and Safety Strategy 2012–2022; however, little progress is evident [78,79]. Farm safety has traditionally not been a high priority of government and industry bodies [79]. While regulation has proven successful in other dangerous industries (e.g., mining and construction) [80], enforcement in agriculture has proven challenging. Within the farming industry, differences in regulations exist between commodities [81]. For example, the horticulture, cotton and dairy sectors have higher health and safety performance when compared to other agricultural sectors (e.g., sheep and beef) as they must follow more stringent quality assurance measures [81]. Regardless of the presence of similar dangers, small/medium sized farms are less likely to engage in safety measures to protect children than larger farms [37]. Socio-economic status may contribute to this lower engagement. As most farms are small businesses—with the workplace also the home/private property—farmers may view regulations as optional and an infringement on their personal home and private property. Voluntary safety standards and common sense are relied on to prevent injury [16,82]. 

While international literature has described children’s involvement in farming tasks [83,84,85], the roles performed by Australian children are less understood. Kennedy and colleagues [86] identified Australian children as actively involved in farming; commonly engaging with machinery, vehicles, firearms and livestock. These tasks were normalized, considered fun, and identified as activities that families can do together. The presence of family members and children as workers is a distinguishing feature of the agricultural industry [56]. Agriculture accounts for 71% (108 million) of the world’s child labour [9]. The Food and Agriculture Organization of the United Nations [9] defines child labour as *“work that is inappropriate for a child’s age, affects children’s education, or is likely to harm their health, safety or morals”*. 

Child labour is a multifaceted issue, influencing schooling, healthcare, labour market conditions, social protection, basic services, social norms and cultural practices [87]. Most child labour is non-paid informal work for the family, and varies around the world from deliberate exploitation to more subtle methods commonly seen in Australia—such as children staying home from school to assist during shearing. While parents may not consider this child labour, it has the potential for negative health and education consequences [87]. Research is yet to explore the full economic contribution of Australian children to the agricultural industry. 

Despite the known presence of children within the farm workplace, Occupational Health and Safety (OHS) standards do not currently protect them. While a range of laws apply across Australia stipulating the age at which children can commence employment [88,89], most farm work performed by children is unregulated due to the exemption for working in a family business [82,90]. Worksafe Victoria [90] stipulates that children on farms should only complete ‘light work’ that is not likely to harm their health, safety and welfare, and does not restrict their ability to attend school. However, the term ‘light work’ remains vague. 

The Child Employment Act 2004 (Victoria) provides more specific guidance on work that could cause harm to children, including moving vehicles and uncontrolled animals—both of which are prevalent on farms. It is currently unknown whether new workplace manslaughter laws will increase the protection for children assisting on farms; however, the Victorian Farmers Federation were denied an exemption to family farms—therefore, they should be enforced [91]. While legislation may provide some protection to children, there appears to be no examples of prosecution following a child farm-related fatality. Ultimately, parents and guardians are left responsible for judging their child’s involvement in farm tasks [85]. Leaving decision-making regarding child safety at farm workplaces to parents/guardians may not be sufficient protection [52,66]. 

#### 3.3.5. Financial Priorities 

The perceived costs and concerns of reduced productivity may limit the adoption of OHS solutions [16]. Durey and Lower [92] suggested that farmers only spent money on safety if it improved the business’ overall financial position. Investments in safety often compete with investments to increase productivity and general family needs. Typically, farmers choose lower-order, cheaper safety options [93]. Saluja and colleagues [2] explained that parents acknowledge there are changes they can implement to enhance their child’s safety but are faced with limited economic and practical resources. To address this, financial incentives, such as tractor rollover protection rebates, have been effective in the past in increasing investments in safety 37]. The government of the Australian state of Victoria recently (2021) announced the provision of $5000 rebates on capital works and equipment to improve farm safety, inclusive of fencing for safe play areas on farms [94]. 

Pollock [95] estimated the cost of farm-related deaths between 2001 and 2004 to the Australian economy was just over $650 million (based on 2008 dollars). However, this did not take into consideration the costs of non-fatal injuries (hospital costs and time lost from work). Safework Australia [96] identified that agricultural workers required longer periods off work following an injury compared to all other industries. In Australia, employed workers who are injured on farms are eligible for workers’ compensation. However, according to the 2013 Safework Australia report [12], only half of the workers in the agricultural sector were eligible for workers compensation, and further, many that are eligible do not put in a claim. 

Many farmers are self-employed and, therefore, not eligible for workers’ compensation as they are not defined as a worker. These individuals are able to purchase loss of income insurance policies for injury or disability; however, these policies usually do not cover other family members working on the farm, such as children and partners (who are also often not defined as workers). Importantly, the universal healthcare system (Medicare) in Australia does give residents access to health and hospital care at little to no cost [97]. Therefore, investment in farm safety has the potential to result in improved economic outcomes for farmers.

#### 3.3.6. Limited Targeted Farm Safety Programs 

Child farm safety in Australia came under focus in the 1990s following reports of high rates of injury [11]. Previously, the focus of agricultural OHS approaches was on employees of larger enterprises [57]. While Farmsafe Australia promoted interventions targeting child safety on farms (e.g., safe play areas, seatbelts and helmet use), funding ceased in 2006 30]. 

It has been estimated that 82% of child deaths and 58% of child hospitalisations could have been prevented if the Farmsafe strategies were in place [46]. The Rural Injury Prevention Primary Education Resource (RIPPER) was developed in 2005 to align with the school curriculum and educate children on farm safety. However, this program is yet to be evaluated. The farm safety guidelines developed in the late 1990s may still be relevant today, as the major farm hazards have remain unchanged. However, the effectiveness of these guidelines has not been assessed [30]. 

Education—together with culture and behaviour changes—is required for effective prevention of child farm injuries [30,52]. Typically, education programs are considered less effective injury prevention interventions, and their long-term influence is unknown. They should, therefore, be implemented alongside other interventions, such as engineering controls and regulation [30,82]. As farming communities are heterogeneous, tailored prevention programs are required to address specific needs, empower communities to make better health and safety decisions and to create universal action [60]. Children are active agents in their own risk taking behaviour and, likewise, their own injury prevention. Targeting children in prevention efforts has been shown to—in the short term—influence their safety behaviour and that of their parents [70,72]. 

#### 3.3.7. Hierarchy of Control 

**Figure 2:**
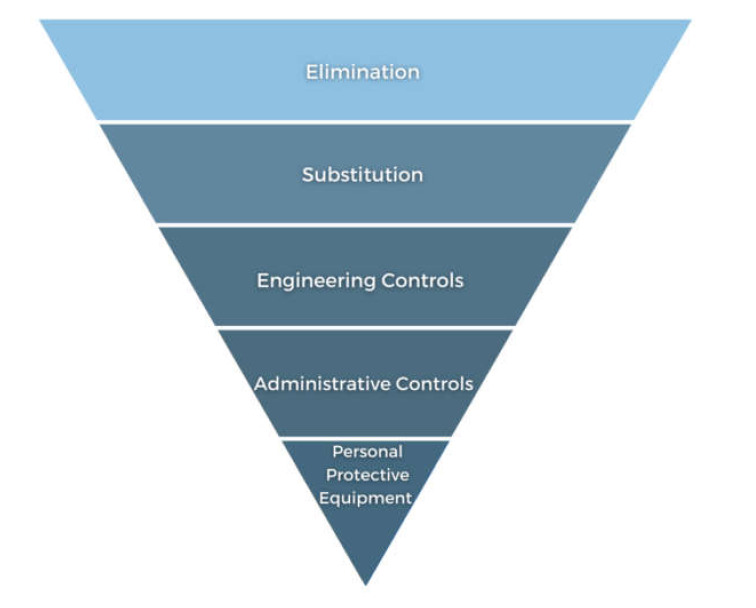
Hierarchy of Control (modified from the National Institute for Occupational Safety and Health) [100].

The hierarchy of control aims to create safer workplace environments by providing a step-by-step ranking approach to eliminate or reduce risks of injury [98,99,100] (Figure 2). Hazard elimination is viewed as the highest level of control in the hierarchy (e.g., filling in old sheep dips or removing keys from vehicles). Further stages are the reduction of risk through substitution (e.g., substitute horse/motorbike with transport appropriate for the child’s size) and engineering controls (e.g., altering motorbike throttle speed). The use of administrative controls (e.g., family rules that everyone must follow) and personal protective equipment (PPE) (e.g., helmets and seatbelts) are the final stages of the hierarchy [98,99]. 

Dosman and colleagues [101] suggested that these steps could be used as a series of options—selected depending on the agricultural task or process involved. Adherence to a variety of the controls is more reliable in reducing injury risk [99,101]. While, the hierarchy has not been evaluated specifically in relation to children on farms, it has been well documented in workplace injury prevention. This may be important, as the elimination of children from the farming workplace is often not possible, and involving the family is strongly engrained within the farming lifestyle. 

Lower and Temperley [79] estimated that 70% of quad incidents could be averted using engineering controls (such as crush protection devices), PPE (such as helmets), and hazard elimination (ensuring children do not ride). Safety campaigns have promoted the need for children to wear safety protection, such as a helmet, when riding horses and motorbikes; the need for seatbelts when driving in a utility around the farm; and features such as a safety switch in the workshop or on machinery seats when a driver is not seated [102]. However, the actual implementation of these is largely unknown, as is the removal rates of these safety switches after purchase. 

While the majority of engineering controls target farming adults, some concentrate on children, for example, throttle adjustment to limit motorbike speed [18]. Limited regulation of the agricultural industry means most engineering controls must be voluntarily adapted [17,82] or legislated—such as the recent Australian Competition and Consumer Commission announcement regarding improved safety requirements for quad bikes manufactured or supplied in Australia [103]. If farmers do not employ workers, they may be exempt/think they are exempt from many workplace rules as they are performing work on private property. Given that the children assisting may not be considered employees, the adoption of engineering controls may not be compulsory. 

PPE can protect the body from injury. However, as seen in the hierarchy of control, this is the lowest level of control. There is also limited understanding of the utilisation of the wide range of PPE available to children on farms. National coronial evidence highlighted that only 20–25% of children who died in a quad bike fatality were wearing helmets [47,50]. A higher rate of helmet use was identified among non-fatal injuries, with 36% of hospitalisations noting the use of helmets [48]. While this suggests the protective influence of helmets against more serious injury, the evidence was based on data from only one hospital, and may not be generalisable. Self-report surveys of farmers in New South Wales identified higher rates of helmet use—38% of quad and 46% of motorcycle riders, and 73% of horse riders [37]. However, self-report responses are vulnerable to participants providing socially desirable answers. 

#### 3.3.8. Supervision 

Supervision is the most common method of protecting children from danger. Young children require constant supervision with a safe play area and rules, while observation is imperative for older children performing developmentally appropriate tasks [46,90]. However, inadequate supervision is a leading cause of child farm injury [54]. Over half of Australian child farm deaths between 2001 and 2019 had no active supervision [30]. Active supervision is described as specific and overt behaviours by parents/supervisors with children, such as scanning, escorting and interacting, to prevent problem behaviour [104]. 

Simultaneously performing farm work and providing effective supervision is challenging [105]. Stiller and colleagues [46] identified that 58% of farmers with children living on, or regularly visiting their farm, provided care while in the farming workplace. Only 30% of these farmers acknowledged that this was due to limited access to childcare services, suggesting that many parents consciously choose to take children into the farm workplace. This may be due to the long work hours, days of work, cost, isolation from family networks, potential travel required to utilize services, or the desire to engage children in the farming lifestyle. 

#### 3.3.9. Safe Play Areas 

Safe play areas are effective safety measures for the prevention of child farm-related injury by providing secure boundaries to contain the movement of young children (under five) into unsafe work areas [42,52,53,105,106]. Safe areas are recommended ahead of other active interventions, such as supervision, as there is less chance of error. However, combining a safe play area with close active supervision has the potential to reduce the risk of child injury across all aspects of the farm [106]. 

Although broadly promoted, research suggests that the uptake of safe play areas is limited [53,81] and varies between commodity groups [25]. Surveys of farmers in the Australian state of New South Wales suggested that only 44% of farms had safe play areas with a fence that would be difficult for a small child to infringe [106]. Further work is required to understand how to engage farmers and identify barriers to normalising and adopting this relatively simple and cost-effective safety measure [53]. 

### 3.4. Limitations of Current Child Farm Safety Literature and Data 

A range of limitations exist in the data used to quantify child farm-related injuries, potentially resulting in conservative statistics. These include:

- The National Farm Injury Data Centre consistently monitors Australian print media for farm-related injuries, providing annual reports on both farm-related fatal and non-fatal injuries [107]. However, farm-related injuries are not always published in the media, especially in the case of non-fatal injuries.
- Child farm-related injuries/fatalities are only captured from hospital and coronial records—less severe injuries are missed (e.g., attendance at General Practitioner clinics or bush nursing centres) [30].
- Hospital and coronial records are collected differently between jurisdictions and so are likely to capture varied data types. Additionally, these records are not developed for the purpose of injury research.
- The process of data linking is vulnerable to errors [41].
- Injury data does not consistently capture occupation or place of injury, resulting in records not including farm identifiers.
- Reliance on self-reporting farm residence can be subjective.
- The number of children living on—or visiting—farms remains unreported, resulting in challenges determining the rate of children injured on farms.
- Child farm injuries are not always accurately reported, as children helping out in the family business are not classified as employees and, consequently, not captured in workers compensation data. Data accuracy is reliant on parents reporting their child’s activity at the time of injury. Parents may ‘hide’ injuries or describe them as ‘recreational incidents’, not as workplace accidents.

## 4. Discussion

The reviewed literature provides a foundation for the understanding of child farm-related injuries in Australia. It is apparent the representation of children in farm-related injury statistics has remained consistent over the last decade [30,39]. Furthermore, the literature is in agreement on the key farming hazards that invariably cause these injuries. Although these hazards (water bodies, quad bikes, motorbikes, utility vehicles/cars, tractors and horses) have been well-identified, they remain the key sources of child farm-related injury [30]. This review also identified factors noted in the literature as contributing to the increased vulnerability of children on farms. 

It is important to understand the limitations in the current literature and key gaps in knowledge. To date, Australian research on child farm-related injury has largely been quantitative in design, resulting in little understanding of typical family farming behaviours. As this review highlights; it is known that children experience injury on farms, and the key agents responsible for these injuries are well-identified. However, research is yet to explore children’s level of exposure to these farming hazards. Furthermore, the typical attitudes, behaviours and actions of both children and parents on farm safety remain unknown. In the early 2000s, Marlenga and colleagues [84] highlighted this, acknowledging that patterns of childhood injury were frequently interpreted without an understanding of their level of exposure to occupational risk. In order to prevent these injuries from happening, an understanding of the social context of child farm-related injuries is required. 

Research suggests that farmers are often willing to share their stories to minimise the risk for others [108]. Absent from the literature is the exploration into the lived experiences of child farm-related injury. First-hand experiences of injury—if conveyed appropriately—can provide an invaluable source of knowledge. While an emotive topic, lived experience can be used to increase the understanding of context and long-term consequences of injury and inspire positive action to reduce risks of farm-related injury. Typically, farmers view their peers as relatable and trustworthy, with narrative-based stories from fellow farmers being more influential than statistics [108]. Farm-related injury can have lasting effects on the mental wellbeing of victims as well as family members [109]. Farmers can hold attitudinal barriers that undermine changes in beliefs regarding the adoption of safer farming practices [2]. In order to achieve a behaviour change, firstly, a desire for change is required as well as ensuring farmers have the capacity and information required to take action [110]. As identified in the review, evaluation is yet to be conducted on the previously identified intervention strategies [11,30]. Evaluating these may provide insight into the social and behavioural determinants influencing parents’ decision making in regard to their child’s farm safety. Lack of adaption may potentially indicate a lack of concern or knowledge. Furthermore—and given that injury rates remain steady—understanding the rates and patterns of implementation of farm safety measures would assist in identifying whether these previous interventions were inappropriate, ineffective, or not widely implemented. This is imperative in guiding the development of future prevention strategies. Summers and colleagues [74] described that, in order to develop effective farm safety programs, both parents and children need to be involved. Ehrlich and colleagues [111] further explained this need as they matched parent and child surveys on their knowledge, habits and attitudes around safety behaviours and identified parental reports on their children’s behaviours as often inaccurate. 

Aside from three studies in the early 2000s, engagement with children in farm safety research in Australia has been minimal [70,72,112]. The behaviours and attitudes of children and adolescents influence not only their immediate health, but also their health and wellbeing into adulthood. Resnick and colleagues [113] reported “*at least 70% of premature adult deaths reflect behaviours started or reinforced during adolescence”* (p. 1565). There is a need to understand child and adolescent health as it can mean improved future health and ultimately economic outcomes, due to enhanced productivity [113]. Injury prevention needs to be viewed as a whole family issue—engaging both parents and children is important in creating long-term change to safety cultures and behaviours on Australian farms. 

## 5. Conclusions

Over the last 20 years, the Australian agriculture industry has increased production, and the predicted future returns remain positive [114]. The majority of agriculture production investment and research has focused on the safety and demands of the consumer, such as the traceability of products for food safety, quality, provenance, authenticity and the animal welfare practices associated with production [115]. A greater focus should be provided on the health and safety of the farming families producing the food and fibre [116]. 

In many ways, the need for improved child farm safety has never been greater [30,117]. Farmsafe Australia [62] recently highlighted farming culture as a key contributing factor to the consistent injury statistics over the last decade, suggesting the need to target the next farming generation. Pickett and colleagues [118] wrote a powerful editorial piece about their work in Canadian coronial courts, describing the desperate need to address the issue of child farm-related injuries:

“*I find that I am angry, but am not sure at whom – at farm parents who expose their children to risks, at a rural society that appears to accept these tragedies as part of their fate, at the coroners and health and safety professionals that have yet to challenge the status quo. This is no longer an academic exercise. Something must change.*” (p. 259)

As Barbara Lee, the Director of the United States National Children’s Center for Rural and Agricultural Health and Safety, highlighted, *“children depend on us—as parents, as farmers, as medical providers and as safety professionals to keep them safe”* [[119], p. 1]. This call to action is equally relevant to child farm safety in Australia. An evolution of farming culture and behaviours/practices to create a desire for change is required for an improvement in farm safety to occur [30]. A greater understanding of the context in which child farm safety occurs is a necessary precursor to such evolution.
